# Adverse metabolic outcomes in the early and late postpartum after gestational diabetes are broader than glucose control

**DOI:** 10.1136/bmjdrc-2021-002382

**Published:** 2021-11-08

**Authors:** Christophe Kosinski, Jean-Benoît Rossel, Justine Gross, Céline Helbling, Dan Yedu Quansah, Tinh-Hai Collet, Jardena J Puder

**Affiliations:** 1 Service of Endocrinology, Diabetes and Metabolism, Lausanne University Hospital and University of Lausanne, Lausanne, Switzerland; 2 Department Woman-Mother-Child, Service of Obstetrics, Lausanne University Hospital and University of Lausanne, Lausanne, Switzerland; 3 Service of Endocrinology, Diabetology, Nutrition and Therapeutic Education, Geneva University Hospitals, Geneve, Switzerland

**Keywords:** gestational diabetes mellitus, metabolic syndrome, obesity, glucose intolerance

## Abstract

**Introduction:**

Gestational diabetes mellitus is associated with an increased cardiovascular risk. To better target preventive measures, we performed an in-depth characterization of cardiometabolic risk factors in a cohort of women with gestational diabetes in the early (6–8 weeks) and late (1 year) postpartum.

**Research design and methods:**

Prospective cohort of 622 women followed in a university gestational diabetes clinic between 2011 and 2017. 162 patients who attended the late postpartum visit were analyzed in a nested long-term cohort starting in 2015. Metabolic syndrome (MetS) was based on the International Diabetes Federation definition, and then having at least two additional criteria of the MetS (blood pressure, triglycerides, high-density lipoprotein (HDL) cholesterol, plasma glucose above or below the International Diabetes Federation cut-offs).

**Results:**

Compared with prepregnancy, weight retention was 4.8±6.0 kg in the early postpartum, and the prevalence of obesity, pre-diabetes, MetS-body mass index (BMI) and MetS-waist circumference (WC) were 28.8%, 28.9%, 10.3% and 23.8%, respectively. Compared with the early postpartum, weight did not change and waist circumference decreased by 2.6±0.6 cm in the late postpartum. However, the prevalence of obesity, pre-diabetes, MetS-WC and MetS-BMI increased (relative increase: 11% for obesity, 82% for pre-diabetes, 50% for MetS-WC, 100% for MetS-BMI; all p≤0.001).

Predictors for obesity were the use of glucose-lowering treatment during pregnancy and the prepregnancy BMI. Predictors for pre-diabetes were the early postpartum fasting glucose value and family history of diabetes. Finally, systolic blood pressure in pregnancy and in the early postpartum, the 2-hour post oral glucose tolerance test glycemia and the HDL-cholesterol predicted the development of MetS (all p<0.05).

**Conclusions:**

The prevalence of metabolic complications increased in the late postpartum, mainly due to an increase in fasting glucose and obesity, although weight did not change. We identified predictors of late postpartum obesity, pre-diabetes and MetS that could lead to high-risk identification and targeted preventions.

**Significance of this study**

**What is already known about this subject?**

- Gestational diabetes is associated with an increased prevalence of postpartum glucose intolerance and other cardiovascular risk factors.

**What are the new findings?**

We observed in a multiethnic prospective cohort of women with gestational diabetes:

- Even if waist circumference decreased from early to late postpartum, there was an increase in the prevalence of pre-diabetes, obesity and the metabolic syndrome.
- Different predictors for pre-diabetes, obesity or metabolic syndrome in the late postpartum diabetes were identified.

**How might these results change the focus of research or clinical practice?**

- Identification of predictors of late postpartum pre-diabetes, obesity and the metabolic syndrome could lead to high-risk identification and targeted preventions in women with gestational diabetes.

## Introduction

Gestational diabetes mellitus (GDM) affects 4%–11% of all pregnant women,^1 2^ with a prevalence of 10.9% in women living in Switzerland.^2^ GDM is associated with neonatal complications such as increased birth weight and hypoglycemia, but also with an increased risk of later adverse metabolic health outcomes for the mother, including an up to a seven-fold increase in the future risk of diabetes in the postpartum (PP) period.^3 4^ PP weight retention is a major risk factor for future diabetes development.^5^ International guidelines recommend regular screening (every 1–3 years) for glucose intolerance in PP to prevent and implement early treatment of diabetes to reduce the burden of later complications.^6^

In addition to the increased risk of diabetes, the development of future cardiometabolic complications in women with GDM requires particular attention. GDM is linked to a two-fold risk of coronary artery calcification^7^ or future cardiovascular (CV) events^8^ independently of the development of diabetes.^7 8^ Earlier studies reported a 15%–20% increase in the prevalence of the metabolic syndrome (MetS) in the early PP (6–8 weeks) and up to 40%–50% in the later years.^9–15^ The presence of MetS is associated with a 3–4 fold increased risk of CV disease and all-cause mortality.^16^ Published data are often limited to the evolution of MetS as a whole entity, without mentioning its components or the trajectory of specific body mass index (BMI) categories. MetS is defined by the presence of central obesity and elevated fasting plasma glucose (FPG), blood pressure and lipid profile.^17 18^ Thus, an exhaustive metabolic evaluation during and after pregnancy is necessary to develop targeted interventions in this at-risk population. Previous studies focused either on the MetS, or on some of its components, on a specific ethnic population, or on a single PP timepoint.^11 13 15 19–23^ An exhaustive evaluation of MetsS including also its predictors is increasinlgy important to reduce the CV burden of these women.

This study assessed the changes in weight and BMI categories between prepregnancy, the early (6–8 weeks) and the late (1 year) PP in a real-life clinical ethnically diverse population of women with GDM. In a nested long-term cohort (1-year PP), we also evaluated the changes in prevalence of pre-diabetes and MetS as well all of its components in the early and late PP to identify their long-term predictors.

## Methods

### Study population

**Figure 1:**
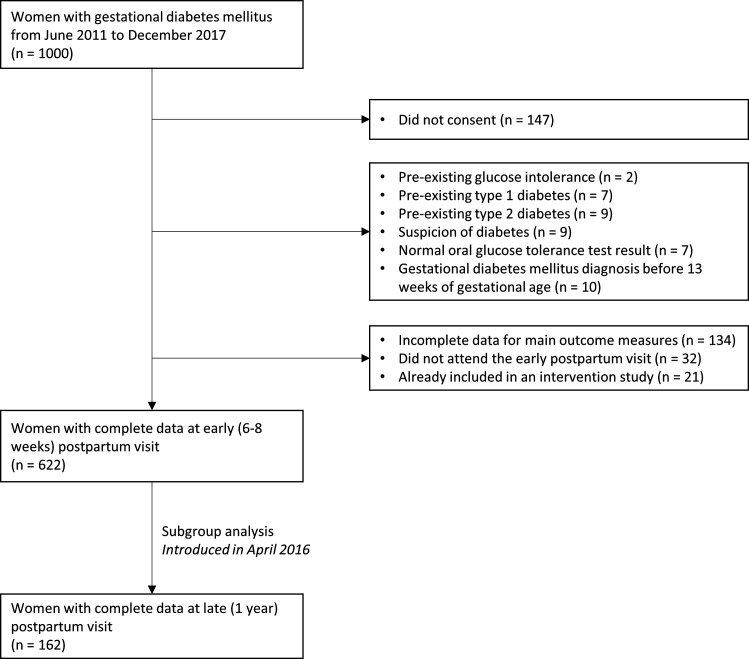
Flow chart.

This is a prospective cohort of women followed at the GDM outpatient clinic of the Lausanne University Hospital and enrolled between June 2011 and December 2017. One thousand women were invited to participate and 147 did not consent (figure 1). Data from this cohort have been previously described elsewhere.^24–29^ We included pregnant women diagnosed with ‘classical’ GDM according to the International Association of the Diabetes and Pregnancy Study Groups and the American Diabetes Association.^6 30^ The exclusion criteria were a pre-existing glucose intolerance, type 1 or 2 diabetes mellitus, a suspicion of diabetes, a normal oral glucose tolerance result, a GDM diagnosis before 13 gestational weeks (total n=44). Women who had missing data on the main outcomes such as MetS, those who did not attend the early PP visit and women already included in an intervention trial (total n=187) were also excluded. After these exclusions, the data of 622 women were used for the final analysis. Women who attended the late (1 year) PP follow-up visit (n=162) were specifically analyzed in an additional nested long-term cohort (figure 1). The number of women is lower for this nested analysis, as the 1-year visit was introduced in August 2015, while the main cohort (pregnancy and 6–8 weeks PP) started in June 2011.

At the first clinical visit, all women were seen by a nurse specialized in GDM or a medical doctor where they received information about GDM and how to self-monitor capillary blood glucose. They also met a dietician one week later, providing them with recommendations on GDM, lifestyle management and weight gain during pregnancy. Regular physical activity was encouraged. According to the international and local guidelines (Vaud Cantonal Diabetes Program),^31^ women were asked to check the capillary glucose value four times per day: fasting in the morning and 2 hours after each meal. During follow-up visits, when glucose values remained above targets at least twice a week during two weeks (fasting blood glucose (FBG) >5.3 mmol/L, and 2-hour postprandial glucose >7 mmol/L), metformin or insulin treatment was initiated, depending on glucose values, patients characteristics and preference. Short-acting insulin analogues were introduced and adapted to achieve 2-hour postprandial glucose ≤7 mmol/L and long-acting insulin analogues to achieve FBG ≤5.3 mmol/L.^6 31^

### Data collection

Glucose tolerance was evaluated with a FPG or a standard 75g-OGTT, before the first GDM visit at the obstetrics hospital outpatient clinic or by their private obstetrician. During the first GDM visit, information on patients’ characteristics including age, educational level, nationality, family history of type 2 diabetes, history of previous GDM, physical activity and smoking during pregnancy were obtained during a structured face-to-face interview. Information on GDM treatment during pregnancy (use of insulin and/or metformin) were obtained from medical charts. In the routine clinical visit at 6–8 weeks’ PP, information about breast feeding and contraception use was recorded. Prepregnancy weight was taken from medical charts or, if missing, was self-reported (for the 1–2 months before pregnancy). We measured weight at the first GDM visit, at the end of pregnancy, at 6–8 weeks and at 1-year PP to the nearest 0.1 kg in light clothes and no shoes with a calibrated stapediometer (Seca). We measured height at the first GDM visit to the nearest 0.1 cm with a regularly calibrated Seca height scale. Glycated hemoglobin (HbA1c) was measured at the first and last GDM visit using the chemical photometric method (conjugation with boronate; Afinion). In the PP visits, HbA1c was measured using a high-performance liquid chromatography method (HPLC).^31^ This method is traceable to the International Federation of Clinical Chemistry and Laboratory Medicine and the Diabetes Control and Complications Trial reference methods. The chemical photometric method (Afinion analyzer) has similar accuracy and precision with the HPLC method.^32 33^ Blood pressure was measured during all visits, after at least 15 min resting sitting (HEM-907, Omron, Japan). FPG and blood lipids were analyzed at early and late PP visits. In the early PP visit, the 75g-OGTT was repeated to measure FPG and 2-hour glucose.^6^

Well-being was assessed by the World Health Organisation (WHO)-5 questionnaire on subjective well-being available in a large variety of languages to accommodate the ethnical diversity of our patients.^34^ This questionnaire was filled at the first GDM clinic visit, at the end of pregnancy and at 6–8 weeks PP. The five items are measured on a 5-points Likert scale ranging from 0 ‘*at no time*’ to 5 ‘*all of the time*’. The final score is then calculated by multiplying the total score by 4, thus ranging from 0 to 100. The questionnaire has adequate validity as an outcome measure in clinical studies.^34 35^ Depressive symptoms were assessed by the Edinburgh Postnatal Depression Scale using a cut-off value ≥13 for symptoms of depression,^36^ at the first GDM clinic visit, at the end of pregnancy and at 6–8 weeks PP.

### Definition of glycemic status, obesity, and metabolic syndrome

For the PP visits, pre-diabetes was defined by a FPG ≥5.6 mmol/L to <7 mmol/L, and/or an HbA1c ≥5.7%–<6.5%, and/or a 2-hour OGTT plasma glucose ≥7.8 mmol/L–<11.1 mmol/L; diabetes was defined with a FPG ≥7 mmol/L and/or an HbA1c ≥6.5% and/or 2-hour OGTT plasma glucose ≥11 mmol/L.^37^ Obesity was defined as BMI ≥30 kg/m^2^. We followed the MetS definition of the International Diabetes Federation (IDF) based on the presence of central obesity (either waist circumference >80 cm in women or BMI ≥30 kg/m^2^) and any two of the following criteria: triglycerides ≥1.7 mmol/L, high-density lipoprotein (HDL)-cholesterol <1.3 mmol/L, blood pressure ≥130/85 mm Hg, FPG ≥5.6 mmol/L or type 2 diabetes.^17 18^ Both criteria for central obesity were used (referred to as MetS-WC and MetS-BMI), as waist circumference (WC) might not be accurate and can change in the early PP period. PP weight retention was defined as the weight retained in PP compared with the prepregnancy weight.

### Statistical analyses

Sociodemographic, clinical and biochemical characteristics are presented as mean±SD for continuous variable, or number of patients (percentages) for categorical variables. Changes in weight were presented as mean±SD of the individual weight difference. Changes in differences between continuous variables at two time points were assessed with paired t-test or by χ^2^ test for categorical variables. When appropriate the results are presented as mean with 95% CI. Comparison of continuous variables at more than two time points was performed using pairwise comparison of means (post hoc Tukey’s test) and are presented in the text as mean difference (95% CI) or OR (95% CI). The overall comparison between the time points is based on an analysis of variance.

The variables used as potential predictors of obesity, pre-diabetes or MetS in the late PP (long-term nested cohort) were first assessed in a univariate logistic regression. Factors with a p value ≤0.20 in the univariate analysis were then included in a multiple logistic regression analysis with backward stepwise model selection, where all analyses were adjusted for maternal age. In an additional analysis, we focused only on non-metabolic factors (ie, variables not included in the definition of the MetS) to assess potential predictors beyond the purely metabolic factors. This was based on our assumption that metabolic predictors could best predict metabolic outcomes and a broadening could help to find additional alternative prevention targets. Details about metabolic and non-metabolic factors are listed in online supplemental table 1.

10.1136/bmjdrc-2021-002382.supp1 Supplementary data

In the sensitivity analysis, missing data were imputed before running the main analyses, using multiple imputation using chained equations. Two-tailed p values <0.05 were considered statistically significant. All analyses were performed using STATA statistical software (V.15.0; Stata Corp).

## Results

The characteristics of the 622 women attending the GDM clinic are described in table 1.

**Table 1:** Descriptive characteristics

	Main cohort (n=622)	Nested long-term cohort (n=162)
Age at first GDM visit (years)	33.2 ± 5.4	33.5 ± 5.5
Nationality		
Switzerland	173 (27.8)	54 (33.3)
Europe and North America	200 (32.1)	52 (32.1)
Africa	106 (17.0)	30 (18.5)
Asia and West Pacific	90 (14.5)	21 (13.0)
Latin America	29 (4.7)	2 (1.2)
Other	24 (3.9)	3 (1.9)
Educational status	*(n=265)*	*(n=103)*
None	4 (1.5)	3 (2.9)
Mandatory school	62 (23.4)	32 (31.1)
General and vocational education	33 (12.5)	14 (13.6)
High school	56 (21.1)	22 (21.3)
University	110 (41.5)	32 (31.1)
Occupation at first GDM visit	*(n=480)*	*(n=147)*
Student	19 (4.0)	4 (2.7)
Employed	289 (60.2)	90 (61.2)
Unemployed	74 (15.4)	27 (18.4)
At home	98 (20.4)	26 (17.7)
Smoking status at first GDM visit		
Current	85 (13.7)	25 (15.5)
Former*	7 (1.1)	2 (1.2)
Previous pregnancy prior to first GDM visit		
None	194 (31.2)	55 (33.9)
1	178 (28.6)	49 (30.3)
2	126 (20.3)	32 (19.8)
3	70 (11.2)	13 (8.0)
≥4	54 (8.7)	13 (8.0)
History of prior GDM†	*(n=428)*	*(n=107)*
Present	47 (11.0)	14 (13.1)
Family history of diabetes		
First degree‡	202 (32.5)	58 (35.8)
Second degree§	120 (19.3)	29 (17.9)
Psychological status		
WHO well-being score at first GDM visit	60.3 ± 19.9 *(n=300)*	62.6 ± 19.6 *(n=148)*
EPDS at early PP visit	6.0 ± 4.4 *(n=212)*	6.0 ± 4.4 *(n=96)*
Treatment during pregnancy		
Only lifestyle intervention	286 (46.0)	60 (42.0)
Lifestyle intervention+metformin	40 (6.4)	5 (3.1)
Lifestyle intervention+metformin+insulin	13 (2.1)	3 (1.8)
Lifestyle intervention+insulin	283 (45.5)	86 (53.1)
Breastfeeding at early PP visit	*(n=538)*	*(n=151)*
Present	497	138
Contraception at early PP visit	*(n=180)*	*(n=62)*
Present	110	37

Data are presented as mean±SD or as n (%). The number of patients for each analysis is indicated in brackets and in italic, when different from the total number of patients.

*Stopped since the pregnancy.

†Only applicable to the 428 patients with a previous pregnancy.

‡Mother, father, sister and brother.

§Grandmother, grandfather, step sister, step brother, niece and nephew.

EPDS, Edinburgh Postnatal Depression Score; GDM, gestational diabetes mellitus; PP, postpartum; WHO, World Health Organisation.

This was an ethnically diverse population whose medical history highlighted an 11% prevalence of GDM in a previous pregnancy and 52% of family history of diabetes. The mean weight at the first GDM visit was 79.8±15.1 kg, representing a mean weight gain of 10.4±5.5 kg at 27.0±3.5 gestational age compared with the prepregnancy weight (p<0.001, online supplemental table 2). During pregnancy, the treatment of GDM consisted of lifestyle modification for 46.0% of women, metformin only for 6.4% and insulin for 47.6%, either alone or in combination with metformin (table 1).

### Changes in weight and BMI categories

At the early PP visit, women had a PP weight retention of 4.8±6.0 kg (p<0.001) compared with their prepregnancy weight. Particularly, fewer women remained in the normal BMI range (34.1% vs 51.1% before pregnancy), with 8.3% more being now overweight and 8.7% more being obese (p<0.001; table 2).

**Table 2:** Main cohort (6–8 weeks postpartum)

	Before pregnancy (n=622)	Early PP visit (n=622)	P value
Weight (kg)	69.2±15.2	74.0±15.0	<0.001
Waist circumference (cm)	–	93.7±11.3	–
BMI (kg/m^2^)	25.9±5.3	27.7±5.2	<0.001
BMI category (kg/m^2^)			<0.001
Normal (<25.0)	317 (51.1)	212 (34.1)	
Overweight (25.0–29.9)	179 (28.8)	231 (37.1)	
Obesity (≥30.0)	125 (20.1)	179 (28.8)	
Metabolic syndrome			
WC defined	–	148 (23.8)	–
BMI defined		64 (10.3)	
HbA1c			
%	–	5.4±0.4	–
mmol/mol		36.0±4.4	
Pre-diabetes, n (%)	–	180 (28.9)	–

Data are presented as mean±SD or as n (%).

BMI, body mass index; HbA1c, glycated hemoglobin; PP, postpartum; WC, waist circumference.

Within the three obesity categories, all prevalence increased in the PP period (p<0.001, online supplemental table 2).

In the nested long-term cohort, compared with the early PP visit, weight did not decrease in the late PP (−0.4 kg (95% CI −4.2 to 3.5); p=0.97) and thus remained significantly higher than before pregnancy (+4.2 kg (95% CI 0.7 to 8.4); p=0.03; table 3).

**Table 3:** Nested long-term cohort (1-year PP)

	(A) Before pregnancy (n=162)	(B) Early PP visit (n=162)	(C) Late PP visit (n=162)	Overall comparison (p value)	B versus A (p value)	C versus A (p value)	C versus B (p value)
Weight (kg)	70.1±14.2	74.6±13.7	74.1±16.6	0.61	0.016	0.03	0.97
Waist circumference (cm)	–	94.6±9.8	91.8±12.4	–	–	–	<0.001
BMI (kg/m^2^)	26.2±4.9	27.9±4.7	27.7±5.8	0.546	0.008	0.018	0.96
BMI category (kg/m^2^)							
Normal (< 25.0 kg/m^2^)	78 (48.1)	46 (28.4)	64 (39.5)	<0.001	<0.001	<0.001	<0.001
Overweight (25.0–29.9 kg/m^2^)	52 (32.1)	69 (42.6)	46 (28.4)				
Obesity (≥ 30.0 kg/m^2^)	32 (19.8)	47 (29.0)	52 (32.1)				
Metabolic syndrome							
WC defined	–	35 (21.6)	50 (32.1)	–	–	–	<0.001
BMI defined		13 (8.0)	26 (16.0)				
OGTT (mmol/L)							
T0		5.0±0.6	5.5±0.7	–	–	–	<0.001
T120 min		5.3±1.7	–				
HbA1c							
%	–	5.3±0.4	5.4±0.4	–	–	–	0.96
mmol/mol		34.0±4.4	36.0±4.4				
Pre-diabetes	–	42 (25.9)	76 (47.2)	–	–	–	0.001
Blood pressure (mmHg)							
Systolic	–	112.3±11.8	114.4±11.1	–	–	–	0.33
Diastolic		72.0±8.2	72.3±9.4				0.99
Lipids (mmol/L)							
Total cholesterol	–	5.2±1.0	4.4±0.8	–	–	–	<0.001
LDL-cholesterol		3.1±0.9	2.5±0.7				<0.001
HDL-cholesterol		1.5±0.4	1.4±0.4				0.022
Triglycerides		1.3±0.7	1.2±0.7				0.076

Data are presented as mean±SD or as n (%).

BMI, body mass index; HbA1c, glycated hemoglobin; HDL, high-density lipoprotein; LDL, low-density lipoprotein; OGTT, oral glucose tolerance test; PP, postpartum; WC, waist circumference.

Similarly, the mean BMI remained higher than before pregnancy (+1.6 kg/m^2^ (95% CI 0.2 to 2.9); p=0.018). Compared with the early PP, changes in BMI categories showed a mixed picture: the number of women in the normal weight category increased by 11.1%, decreased in the overweight category by 14.2% and increased in the obesity category by 3.1% (all p<0.001). Overall, the prevalence of obesity increased from 19.8% before pregnancy to 32.1% in the late PP (p<0.01) (table 3; online supplemental table 3).

### Changes in prevalence of pre-diabetes and MetS

In early PP, the prevalence of pre-diabetes, MetS-BMI and MetS-WC were 28.9%, 10.3% and 23.8%, respectively (table 2). The prevalence of each single components of MetS using the IDF cut-offs were 30% for low HDL-cholesterol, 23% for high triglycerides, 16% for elevated blood pressure and 13% for increased FPG.

In the nested long-term cohort, WC decreased from early to late PP (−2.6±0.6 cm, p<0.001) and HbA1c remained stable (0.02% (95% CI −0.09 to 0.14); p=0.96; table 3). However, FPG increased by 0.5 mmol/L (95% CI 0.3 to 0.7; p<0.001) and the prevalence of pre-diabetes increased 1.8-fold (from 25.9% to 47.2%, p=0.001). The prevalence of MetS-BMI also increased by two-fold (from 8.0% to 16.7%, p≤0.001) and of MetS-WC by 1.5-fold (from 21.6% to 32.1%, p≤0.001). This increased prevalence of MetS (defined by either BMI or WC) was driven mostly by the increase in FPG, while the other components (HDL-cholesterol, triglycerides, and blood pressure) improved or did not change from early to late PP.

### Predictors of obesity, pre-diabetes, and metabolic syndrome in the late PP

BMI before pregnancy was the only independent significant predictor of obesity in the late PP (OR 1.70, 95% CI 1.40 to 2.07) (table 4). When factors contained in the MetS-WC definition (‘non-metabolic factors’) were removed, the use of glucose-lowering treatment (metformin and insulin) during pregnancy also predicted obesity (table 4).

**Table 4:** Predictors of obesity, pre-diabetes and MetS-WC at 1-year PP*

All factors included	OR (95% CI)	Factors not contained in MetS-WC definition§	OR (95% CI)
Predictors of obesity			
BMI before pregnancy†	1.70 (1.40 to 2.07)	WHO well-being score at 6–8 weeks PP	0.98 (0.96 to 1.00)
Treatment during pregnancy (metformin, insulin)	3.17 (0.93 to 10.8)	Treatment during pregnancy (metformin, insulin)†	2.39 (1.10 to 5.17)
Predictors of pre-diabetes			
BMI before pregnancy	1.06 (0.98 to 1.15)	Family history of diabetes†	2.10 (1.05 to 4.20)
Fasting glucose at 6–8 weeks PP†	3.23 (1.32 to 7.92)	Personal history of GDM	2.82 (0.80 to 9.99)
2-hour plasma glucose at 6–8 weeks PP	1.28 (0.95 to 1.72)	Employed during pregnancy	0.59 (0.29 to 1.19)
HbA1c at 6–8 weeks PP	2.75 (0.85 to 8.84)		
Family history of diabetes	1.88 (0.88 to 4.05)		
Predictors of metabolic syndrome‡			
Systolic blood pressure at end of pregnancy†	1.08 (1.01 to 1.15)	Family history of diabetes	2.15 (0.88 to 5.27)
Systolic blood pressure at 6–8 weeks PP†	1.08 (1.02 to 1.15)		
2-hour plasma glucose at 6–8 weeks PP†	1.59 (1.07 to 2.37)		
HDL-cholesterol at 6–8 weeks PP†	0.05 (0.01 to 0.29)		

*Factors were first selected in univariate analyses, then in a multiple stepwise regression with a cut-off p value ≤0.20. This tables lists only the significant factors after backward selection.

†Significant predictors (p<0.05).

‡Defined by waist circumference.

§Non-metabolic factors are listed in online supplemental table 1.

BMI, body mass index; GDM, gestational diabetes; HbA1c, glycated hemoglobin; HDL, high-density lipoprotein; MetS, metabolic syndrome; PP, postpartum; WC, waist circumference.

Predictors of pre-diabetes were FPG levels in early PP and family history of diabetes; only family history of diabetes in ‘non metabolic factors’ (table 4).

Predictors of MetS-WC were systolic blood pressure at the end of pregnancy and in the early PP, as well as HDL-cholesterol and 2-hour post-OGTT glucose value at the early PP visit; any ‘non metabolic factors’ were statistically significant (table 4).

No collinearity was found between all these predictors (data not shown). In the sensitivity analysis with imputed data, we found similar results for the predictors described previously (data not shown).

## Discussion

In this large ethnically diverse cohort of women with GDM followed in a university hospital by an interprofessional team focusing on lifestyle behavior, we found a high prevalence of obesity, pre-diabetes and MetS that even increased in late PP.

Regarding the weight, women with prior GDM are recommended to return to their pre-pregnancy weight within the first PP year.^38^ However, in our cohort, the PP weight retention remained at 4 kg in the late PP despite the professional advice and a clinical follow-up at 6–8 weeks PP. This correlated with an increased prevalence of obesity in both early and late PP.

Regarding glucose homeostasis, we found a two-fold increase in prevalence of pre-diabetes between the early and late PP despite no change in HbA1c. This was driven by a 0.5 mmol/L increase in FPG in late PP. This rise was correlated with the weight at late PP (rho=0.223; p=0.005), supporting that an excessive weight is associated with, mostly hepatic, insulin resistance. GDM is known to be a risk factor of future glucose intolerance, on the continuous spectrum from pre-diabetes to diabetes. Studies demonstrated that a strict glucose control during pregnancy is associated with a decreased risk of future type 2 diabetes.^39^

Regarding the MetS and its components, pathological values were specifically found for HDL-cholesterol and triglycerides. The prevalence of MetS nearly doubled (both for MetS-BMI and MetS-WC definitions) in early and late PP. Our results are in line with previous, mostly older cohorts, but show a higher MetS prevalence range.^9–14 40^

Because metabolic results were similar at the early PP visit between the main cohort and the subgroup (*data not shown*), we inferred that the long-term results demonstrated with the subgroup of 162 women can be applied to the main cohort. In this context, predictors of pre-diabetes, obesity and the MetS in late PP should be identified in order to tailor future clinical trials and provide tools for targeted interventions.

Our evaluation included both metabolic and non-metabolic factors before and during pregnancy in an exhaustive assessment. Regarding predictors of pre-diabetes, FPG level in early PP was the only significant metabolic predictor, with a 3.2-fold risk increase of pre-diabetes in late PP for every 1 mmol/L increase in FPG. Family history was also highly predictive of pre-diabetes. Although not statistically significant, employment during pregnancy was suggestive of a protective factor. In previous studies, risk factors for diabetes or impaired glucose tolerance were BMI before pregnancy,^41 42^ FPG during pregnancy,^41 43^ an earlier gestational age at the time of GDM diagnosis,^43^ the presence of diabetes-related GAD or IA2 antibodies,^44^ insulin use during pregnancy^44^ or women with more than two prior pregnancies.^44^ A systematic review demonstrated other risk factors such as a non-white ethnicity, advanced maternal age and hypertension during pregnancy.^45^ It may seem obvious that glucose at 6–8 weeks PP is a predictor for glucose at 52 weeks PP. Nevertheless, there can be significant changes of insulin sensitivity and secretion and beta-cell decline between the early and late PP that are not observed the same way in all women and can be independent of their changes in weight.^46 47^ Thus, many patients can indeed convert from normal to impaired glucose tolerance and even vice versa, a phenomenon that is observed more frequently in these first 12 months than later on.^48^

The most important but obvious predictor of obesity in late PP was the weight before pregnancy. The need for medical treatment during pregnancy was another predictor, maybe due to a lifestyle approaches sometimes implemented less vigorously in this subgroup, but also to the anabolic effect of insulin.

Due to the clustering of conditions within the MetS entity, its predictors were broad: a 10 mm Hg increase in systolic blood pressure at the end of pregnancy and early PP visit were associated with an 8% increased risk of MetS at late PP, as well as 1 mmol/L increase in 2 hour glucose value during OGTT at early PP visit, while a 1 mmol/L increase in HDL-cholesterol in early PP was protective. Previous studies exploring predictors of MetS are scarce: prepregnancy overweight or obesity, pregnancy systolic blood pressure and need for insulin or metformin treatment were associated with the presence of MetS in GDM women.^11^ GDM itself is known to be a risk factor for future metabolic outcomes, such as central obesity, hypertriglyceridemia, high blood pressure, and with an increased CV risk.^15 16 39^ In our multivariate analyses, and in contrast to Noujah *et al*,^11^ MetS was not predicted by increased weight, but rather by a dysmetabolic context, namely systolic blood pressure, glucose, and lipids. In our evaluation of other factors beyond the metabolic factors, lifestyle habits, such as physical activity and tobacco consumption, were not significantly associated with the development of pre-diabetes, obesity, or MetS. However, a small number of observations, some misreporting and some methodological limitations (only two questions to assess physical activity were used) could explained the difference.

Breast feeding did not predict any metabolic outcomes occurrence with our analysis, which is not in line with previous studies reporting a protective effect.^49^ As 92.4% of our women breast feed in the early PP, this variable might not help enough to differentiate between people or its impact might have been diluted by more relevant predictors.

Glucose intolerance is a known long-term complication of GDM; however, as normoglycemia does not decrease the risk of future CV disease in women with GDM,^7 8^ to focus only on glucose control may let clinician miss essential other CV risk factor. Our results showed also an adverse evolution of other metabolic outcomes, that is, increased prevalence of obesity and MetS in both early and late PP. Interventions during and after pregnancy for patients with GDM should aim at multiple targets, but not limited to optimal glycemic control and weight loss, both in the detection and intervention on these different risk factors. Thus, multitargeted interventions should be tested in future studies, similar to those advocated for the treatment of diabetes. Based on previous study, interventions targeting the behavior of both women and spouses could be necessary in order to alleviate adverse outcomes, based on a social clustering of metabolic traits among individuals.^50^

The strengths of this study are the prospective cohort design, the continuous and repeated evaluation of the patients, the diversity of our population, the analysis of many predictors of major metabolic outcomes in the PP period and the identification of multiples metabolic parameters for potential clinical interventions, including a longer detailed follow-up to late PP. In previous studies, the population were not heterogeneous, sometimes with only one ethnicity studied, but our cohort is composed of diverse ethnicities, which increases the external validity and generalisability of our findings. As table 1 shows, we have Caucasian, black, Asians and Hispanic women included, which all have different risks for obesity, diabetes, and CV disease. However, in many areas of the world as in Switzerland, many women have now mixed ethnicities in various ‘percentages’ and thus an exact relationship between ethnicity and outcome would be difficult to be drawn.

The study also has some limitations. The weight before pregnancy was self-reported in almost half of the patients and therefore could lead to some misclassification. However, the correlation between BMI before pregnancy and at the first GDM visit was high (rho=0.93). In addition, the long-term data were only available in a subgroup of 162 women. Although this was due to changes in our clinical appointment structure, we cannot exclude a bias, but comparison of groups showed similar baseline characteristics (data not shown). To further analyze the evolution of CV risk factors during and after pregnancy, prepregnancy lipid and glucose profile as well as a longer detailed follow-up would have been an interesting addition to our analyses.

## Conclusion

Women with prior GDM had a high risk of later metabolic diseases, including glucose intolerance, obesity, and MetS at late PP. Compared with prepregnancy values, BMI and weight increased substantially in early PP and did not further decrease at late PP leading to an increasing prevalence of obesity. Compared with early PP, the prevalence of MetS and pre-diabetes increased dramatically at late PP. Based on our results, a screening at 1-year PP is absolutely essential. We also identified factors before and during pregnancy as well as in the early PP period that were predictive of later development of MetS, obesity, or pre-diabetes at late PP. Targeted interventions on these factors should be implemented early in standard medical care and should be evaluated in future intervention trials.

## Data Availability

All data relevant to the study are included in the article or uploaded as supplementary information.
