# Supplementary material for: Adverse metabolic outcomes in the early and late postpartum after gestational diabetes are broader than glucose control
Source: BMJ Open Diabetes Res Care. 2021 Nov 8;9(2):e002382. doi: 10.1136/bmjdrc-2021-002382 (PMC8576469; doi:10.1136/bmjdrc-2021-002382)
Supplement: Supplementary data [file bmjdrc-2021-002382supp001.pdf]

Supplementary Appendix.

Supplementary Table 1: Factors used for evaluation of predictors of obesity, prediabetes and MetS-WC at 1 year postpartum

Factors extracted with a p-value ≤0.20 in a multiple stepwise regression
BMI before pregnancy/at the end of pregnancy/at 6-8 weeks PP
Waist circumference 6-8 weeks PP
Gestational weight gain
Fasting glucose at GDM diagnosis/at 6-8 weeks PP
Glycemia t60 at GDM diagnosis
Glycemia t120 at GDM diagnosis/at 6-8 weeks PP
HbA1c at first GDM visit/at the end of pregnancy/at 6-8 weeks PP
Systolic blood pressure at first GDM visit/at the end of pregnancy/at 6-8 weeks PP
Diastolic blood pressure at first GDM visit/at the end of pregnancy/at 6-8 weeks PP
LDL-cholesterol at 6-8 weeks PP
HDL-cholesterol at 6-8 weeks PP
Triglycerides at 6-8 weeks PP
WHO-score at first GDM visit/at the end of pregnancy/at 6-8 weeks PP
EPDS at first GDM visit/at 6-8 weeks PP
History of psychological illness at first GDM visit
Nationality at first GDM visit, professional status at first GDM visit
Smoking status at first GDM visit
Physical activity before pregnancy/during pregnancy
Familial history of diabetes at first GDM visit
History of gestational diabetes at first GDM visit
Previous pregnancy at first GDM visit
Treatment during pregnancy (metformin and/or insulin)
Breastfeeding at 6-8 weeks PP
Contraception at 6-8 weeks PP

“Non metabolic” factors
WHO-score at first GDM visit/at the end of pregnancy/at 6-8 weeks PP
EPDS at 6-8 weeks PP
Nationality at first GDM visit
Professional status at first GDM visit
Smoking status at first GDM visit
Physical activity before pregnancy/during pregnancy
Familial history of diabetes at first GDM visit
History of gestational diabetes at first GDM visit
Previous pregnancy at first GDM visit
Treatment during pregnancy (metformin and/or insulin)
Breastfeeding at 6-8 weeks PP
Contraception at 6-8 weeks PP

Abbreviations: BMI, body mass index; EPDS, Edinburgh postnatal depression score; GDM, gestational diabetes; HDL, high-density lipoprotein; LDL, low-density lipoprotein; MetS, metabolic syndrome; OR, odd ratio; PP, postpartum; WHO, world health organisation.

Supplementary Table 2: Detailed descriptive data of the main cohort.

	**Before pregnancy (n = 622)**	**First GDM visit (n = 605)**	**End of pregnancy (n=556)**	**Early PP visit (n = 622)**
Weight (kg)	69.2 ± 15.2	79.8 ± 15.1	82.0 ± 14.9	74.0 ± 15.0
Waist circumference (cm)	-	-	-	93.7 ± 11.3
BMI (kg/m^2)	25.9 ± 5.3	29.8 ± 5.2	30.7 ± 5.1	27.7 ± 5.2
BMI category (kg/m^2)				
Normal (< 25.0)	317 (51.1)	-	-	212 (34.1)
Overweight (25.0-29.9)	179 (28.8)			231 (37.0)
Obesity class I (30.0-34.9)	91 (14.7)			124 (19.9)
Obesity class II (35.0-39.9)	22 (3.5)			38 (6.1)
Obesity class III (≥ 40.0)	12 (1.9)			17 (2.7)
Metabolic syndrome				
WC-defined	-	-	-	148 (23.8)
BMI-defined				64 (10.3)
OGTT (mmol/L)				
T0	-	5.2 ± 0.7	-	5.0 ± 0.5
T60		9.7 ± 1.9 *(n=473)*		-
T120		7.9 ± 1.9 *(n=496)*		5.5 ± 1.7
HbA1c		*(n=598)*	*(n=266)*	
%	-	5.5 ± 0.4	5.5 ± 0.4	5.4 ± 0.4
mmol/mol		37.0 ± 4.4	37.0 ± 4.4	36.0 ± 4.4
Prediabetes, n (%)	-	-	-	180 (28.9)
Blood pressure (mmHg)		*(n=557)*	*(n=517)*	
Systolic	-	114.5 ± 11.0	115.8 ± 11.5	113.9 ± 12.8
Diastolic		71.8 ± 9.0	74.2 ± 9.8	73.8 ± 9.9
Lipids (mmol/L)				
Total cholesterol	-	-	-	5.2 ± 1.0
LDL-cholesterol				3.1 ± 0.9
HDL-cholesterol				1.5 ± 0.4
Triglycerides				1.3 ± 0.8

Data are presented as mean ± SD, or as n (%). The number of patients for each analysis is indicated in brackets and in italic, when different from the total number of patients. Abbreviations: BMI, body mass index; GDM, gestational diabetes mellitus; HDL, high-density lipoprotein; LDL, low-density lipoprotein; OGTT, oral glucose tolerance test; PP, postpartum; SD, standard deviation; WC, waist circumference.

Supplementary Table 3: Detailed descriptive data of the long-nested cohort.

	Before pregnancy (n = 162)	First GDM visit (n = 157)	End of pregnancy (n = 146)	Early PP visit (n = 162)	Late PP visit (n = 162)
Weight (kg)	70.1 ± 14.2	80.6 ± 14.6	82.9 ± 13.8	74.6 ± 13.7	74.1 ± 16.6
Waist circumference (cm)	-	-	-	94.6 ± 9.8	91.8 ± 12.4
BMI (kg/m^2)	26.2 ± 4.9	30.2 ± 5.0	31.1 ± 4.8	27.9 ± 4.7	27.7 ± 5.8
BMI category (kg/m^2)					
Normal (< 25.0)	78 (48.1)	-	-	46 (28.4)	64 (39.5)
Overweight (25.0-29.9)	52 (32.1)			69 (42.6)	46 (28.4)
Obesity class I (30.0-34.9)	25 (15.4)			34 (21.0)	32 (19.7)
Obesity class II (35.0-39.9)	4 (2.5)			11 (6.8)	15 (9.3)
Obesity class III (≥ 40.0)	3 (1.9)			2 (1.2)	5 (3.1)
Metabolic syndrome					
WC-defined	-	-	-	35 (21.6)	50 (32.1)
BMI-defined				13 (8.0)	26 (16.7)
OGTT (mmol/L)					
T0	-	5.3 ± 1.0 *(n=157)*	-	5.0 ± 0.6	5.5 ± 0.7
T60		9.7 ± 2.2 *(n=121)*		-	-
T120		7.8 ± 2.0 *(n=122)*		5.3 ± 1.7	-
HbA1c			*(n=122)*		
%	-	5.5 ± 0.4	5.5 ± 0.4	5.3 ± 0.4	5.4 ± 0.4
mmol/mol		37.0 ± 4.4	37.0 ± 4.4	34.0 ± 4.4	36.0 ± 4.4
Prediabetes	-	-	-	42 (25.9)	76 (47.2)
Blood pressure (mmHg)					
Systolic	-	113.7 ± 10.2	115.2 ± 10.3	112.3 ± 11.8	114.4 ± 11.1
Diastolic		71.2 ± 8.7	73.8 ± 9.3	72.0 ± 8.2	72.3 ± 9.4
Lipids (mmol/L)					
Total cholesterol	-	-	-	5.2 ± 1.0	4.4 ± 0.8
LDL-cholesterol				3.1 ± 0.9	2.5 ± 0.7
HDL-cholesterol				1.5 ± 0.4	1.4 ± 0.4
Triglycerides				1.3 ± 0.7	1.2 ± 0.7

Data are presented as mean ± SD, or as n (%). The number of patients for each analysis is indicated in brackets and in italic, when different from the total number of patients. Abbreviations: BMI, body mass index; GDM, gestational diabetes mellitus; HDL, high-density lipoprotein; LDL, low-density lipoprotein; OGTT, oral glucose tolerance test; PP, postpartum; SD, standard deviation; WC, waist circumference.
